# DIA-Based Proteomic Analysis of Plasma Protein Profiles in Patients with Severe Acute Pancreatitis

**DOI:** 10.3390/molecules27123880

**Published:** 2022-06-17

**Authors:** He Li, Yansong Xu, Xin Zhou, Taiyang Jin, Ziru Wang, Yuansong Sun, Haiping Wang, Datong Jiang, Chunlin Yin, Bing Shen, Kai Song

**Affiliations:** 1 Department of Emergency, The Second Affiliated Hospital of Anhui Medical University, Hefei 230601, China; lihe@ahmu.edu.cn (H.L.); yansongxu@outlook.com (Y.X.); liheahmu@163.com (X.Z.); 13865950491@163.com (T.J.); wangziru0514@163.com (Z.W.); syslt1987@163.com (Y.S.); efy_wanghp@163.com (H.W.); master.jdtong@163.com (D.J.); ycldoctor@163.com (C.Y.); 2 School of Basic Medicine, Anhui Medical University, Hefei 230032, China

**Keywords:** severe acute pancreatitis, pathogenesis, data-independent acquisition, proteomics, biomarkers

## Abstract

Acute pancreatitis (AP) is a pancreatic inflammatory disease that varies greatly in course and severity. To further the understanding of the pathology of AP, we carried out data-independent acquisition-based proteomic analyses using proteins extracted from the plasma of patients with severe acute pancreatitis (SAP) (experimental group) and healthy volunteers (control group). Compared to the control group, there were 35 differentially expressed proteins (DEPs) in the plasma of patients with SAP. Of those, the expression levels for 6 proteins were significantly increased, and 29 proteins were significantly decreased. Moreover, six candidate biomarkers—VWF, ORM2, CD5L, CAT, IGLV3-10, and LTF—were matched as candidate biomarkers of the disease severity of AP. The area under the receiver operating characteristic of 0.903 (95% CI: 0.839, 0.967) indicated that this combination of these six candidate biomarkers had a good prediction accuracy for predicting the severity of AP. Our study provides specific DEPs that may be useful in the diagnosis and prognosis of SAP, which suggests new theoretical bases for the occurrence and development of SAP and offers potential novel treatment strategies for SAP.

## 1. Introduction

Acute pancreatitis (AP) is a pancreatic inflammatory disease, with approximately 20% of patients developing severe acute pancreatitis (SAP) associated with complications, such as extensive pancreatic necrosis, secondary infection of the necrotic pancreatic tissue, hypovolemic shock, or multiple organ dysfunction [1]. Patients with SAP who have persistent organ dysfunction show a high mortality rate (40%) within the first 14 days after diagnosis; currently, no treatment has reduced the mortality rate in these patients [2]. Therefore, treatment for patients with SAP remains limited, largely due to the lack of understanding of the pathogenesis and prevention of SAP [3,4].

Proteomics is a robust method used to determine the protein expression profile in samples. Many studies have applied proteomics to explore potential pathogens, search for biomarkers, and discover the possible therapeutic targets of AP. For example, Fetaud-Lapierre et al. [5] carried out a time-course proteomic analysis using a rigorous rat model with severe acute necrotizing pancreatitis and found that there are many proteins related to the secretory pathway, which may be the key regulatory factor in pancreatic injury. To understand the underlying differences in the proteomic profiles between patients with mild acute pancreatitis (MAP) and SAP, Papachristou et al. [6] used surface-enhanced laser desorption/ionization time-of-flight mass spectrometry to analyze the serum obtained from 21 patients with MAP and 7 patients with SAP. They found that the serum proteomic profiles well-distinguished MAP from SAP. Sun et al. [7] used a mass spectrometry-based proteomic method to identify proteins before and after Qingyi pellet treatment in a rat model of SAP with lung injury. Their group found that Qingyi pellet treatment may ameliorate SAP with lung injury via cumulative or synergistic interactions of multiple components. Compared with proteomic studies of other diseases, there are few such studies examining AP [8,9]. In addition, to our knowledge, there are no reports of proteomic studies comparing samples between patients with SAP and healthy volunteers. 

In the present study, we used a data-independent acquisition (DIA) technique with mass spectrometry to quantify the plasma protein profiles in patients with SAP and healthy volunteers. Based on this powerful method, a high-throughput proteomic map comprising thousands of peptides and hundreds of proteins was constructed. Bioinformatics analyses were carried out to find biomarkers of SAP and crucial signaling pathways associated with the occurrence and development of SAP. We further validated these selected differentially expressed proteins (DEPs) using an enzyme-linked immunosorbent assay (ELISA) in an independent retrospective cohort and evaluated their predictive value for AP using receiver operating characteristic (ROC) curves.

## 2. Results

### 2.1. Baseline Characteristics of Participants

In total, 96 participants were enrolled in this study; of these, 3 healthy volunteers (control group) and 10 patients with SAP (experimental group) participated in this DIA-based proteomic analysis. The remaining 83 patients with AP were used in subsequent validation experiments. The baseline characteristics of these 13 participants are given in Table 1. The fasting blood glucose level (experimental, 9.47 ± 2.25 vs. control, 4.65 ± 0.75 mmol/L; *n* = 13, *p* = 0.005), white blood cell count (experimental, 15.97 ± 5.66 × 10^9^/L vs. control, 5.93 ± 1.48 × 10^9^/L; *n* = 13, *p* = 0.013), and serum amylase level (experimental, 1490.40 ± 873.33 vs. control, 41.33 ± 5.03 U/L; *n* = 13, *p* = 0.001) were significantly lower in the control group than in the experimental group. 

### 2.2. Quality Verification of Extracted Protein

The total proteins in the samples were quantified and then underwent electrophoresis using sodium dodecyl sulfate-polyacrylamide gel electrophoresis (SDS-PAGE), which was followed by Coomassie brilliant blue staining. The quantitative results are shown in Table S1. According to the electrophoresis results (Figure S1), the total proteins in all samples in the molecular weight range of 15–170 kDa were successfully separated without decomposition. High-abundance proteins were also found in all samples. 

### 2.3. Proteomic Data Acquired by DIA

DIA-based proteomic analyses of all depleted plasma samples were carried out. In the present study, 6207 peptides and 774 proteins were recognized, with a false discovery rate (FDR) of 1% (Table S2). To confirm the reproducibility within groups and differences between groups, we conducted partial least-squares regression for the experimental and control groups (Figure S2). This analysis revealed a similar and clear separation between the proteomic profiles of the two groups. A histogram of the frequency distribution of the peptide lengths is provided in Figure S3A. The figure shows a skewed frequency distribution, with most of the matched [10] peptides having a length between 9 and 11 amino acids and 90% of the peptides having a length within 23 amino acids. 

**Figure 1:**
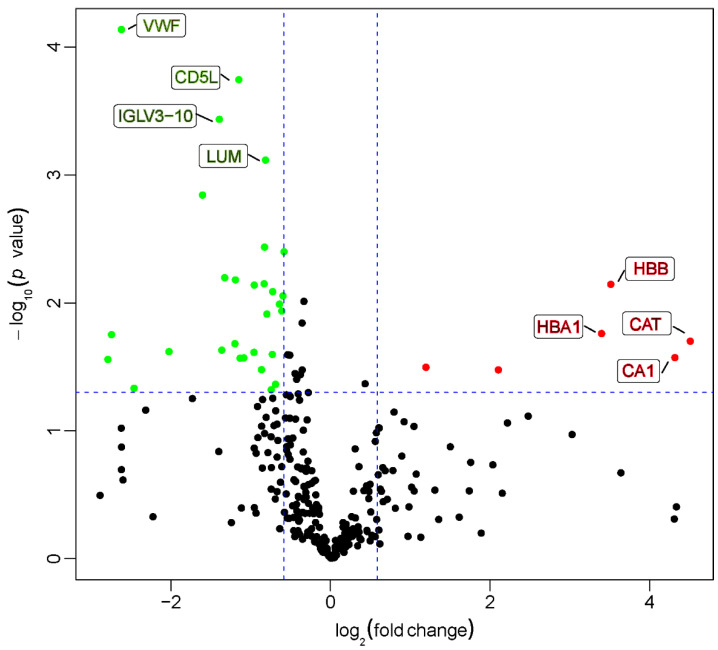
Volcano plot of all proteins. Red dots represent proteins with a significant fold change (FC) ≥ 1.5 (*p* < 0.05); green dots, proteins with a significant FC ≤ 0.667 (*p* < 0.05); black dots, no obvious changes in the protein level.

**Figure 2:**
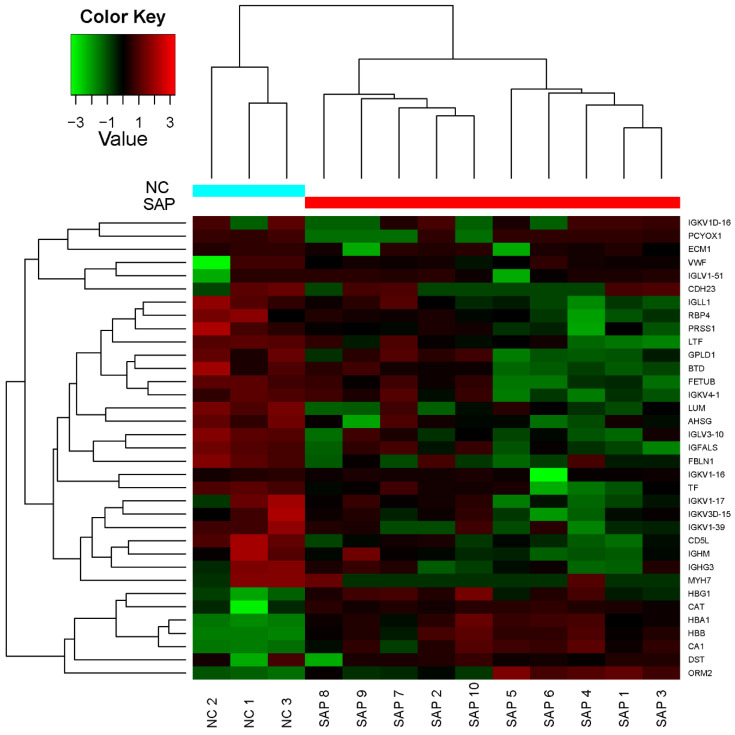
Hierarchical clustering analysis of 35 differentially expressed proteins between the experimental and control groups. Each row in the figure represents a protein, each column is a sample (NC1–NC3 indicates control group healthy volunteers 1–3; SAP1–SAP10, experimental group patients 1–10), and the colors represent different expression levels (the log_2_ values of the quantitative value were obtained, and median correction is shown).

A histogram of the frequency distribution of the log_2_-based fold changes (log_2_ FC) in the ratios of the protein levels between the experimental and control groups is shown in Figure S3B. The histogram indicates that the ratios of most proteins were close to 1. This result suggests that only some proteins showed significant differences between the two groups. To display the correlation between the samples more intuitively, we described the correlations among all samples by using a correlation coefficient matrix (Figure S3C). A volcano plot (Figure 1) was also obtained by plotting log_2_ FC as the abscissa and negative log_10_-based *p* value (−log_10_ *p*) as the ordinate after the proteins in the experimental and control groups were further analyzed using *t*-tests. Compared with the control group, there were 35 DEPs (Table 2), of which, the expression levels of 6 proteins were significantly increased (upregulated proteins), and the expression levels of 29 proteins were significantly decreased (downregulated proteins). In addition, 164 DEPs were found only in the experimental group (termed absent proteins). The hierarchical clustering analysis of 35 DEPs, between the experimental and control groups, showed that the expression patterns in the experimental group clearly differed from those in the control group, and protein expression in each group was clustered (Figure 2). 

### 2.4. Gene Ontology (GO) Functional Annotation and Enrichment Analysis of 35 DEPs

**Figure 3:**
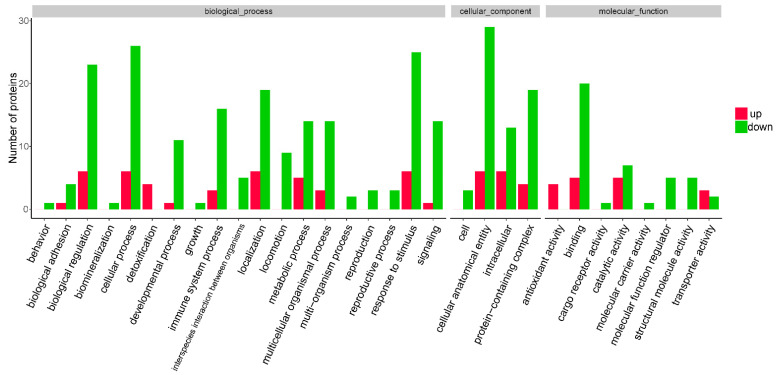
Gene ontology (GO) functional annotation and enrichment analysis of 35 differentially expressed proteins (DEPs) detected between the experimental and control groups. (A) GO functional annotation analysis of 35 DEPs. Up represents proteins that are upregulated between the experimental and control groups; down, proteins that are downregulated.

The role of eukaryotic genes and proteins in cells can be described by GO functional annotation analysis, providing the potential properties of the genes and gene products in organisms. The GO functional annotation analysis results of the 35 DEPs are shown in Figure 3. The term in the biological process category with the highest number of DEP annotations was cellular process (*n* = 32 proteins; with the top three upregulated proteins in this category being CAT, HBB, and HBG1). For the cellular component category, the term with the highest number of DEP annotations was cellular anatomical entity (*n* = 35 proteins; with the top three upregulated proteins in this category being CAT, HBA1, and HBG1). The term in the molecular function category with the highest number of DEP annotations was binding (*n* = 25 proteins; with the top three upregulated proteins in this category being HBB, HBA1, and HBG1). GO functional enrichment analysis can determine the main biological functions of the proteins. The GO functional enrichment analysis results of the 35 DEPs are shown in Figure S4 and indicate that the DEPs were enriched in the terms blood microparticle, immunoglobulin production, and production of molecular mediator of immune response.

### 2.5. Kyoto Encyclopedia of Genes and Genomes (KEGG) Pathway Annotation and Enrichment Analysis of 35 DEPs

**Figure 4:**
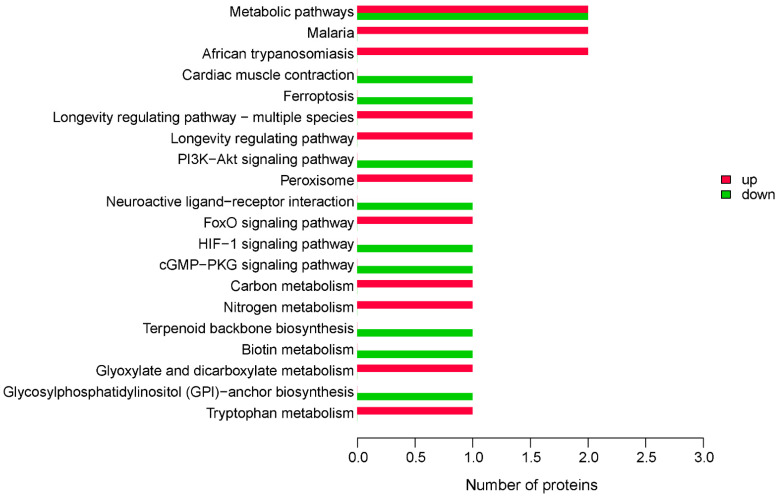
Kyoto Encyclopedia of Genes and Genomes (KEGG) pathway annotation analysis of 35 DEPs. Up represents proteins that are upregulated between experimental and control groups, and down represents proteins that are downregulated.

KEGG pathway annotation analysis of the 35 DEPs (Figure 4) indicated that the DEPs were mainly annotated with the terms metabolic pathways, malaria, and African trypanosomiasis. The downregulated DEPs involved in the metabolic pathways were BTD and GPLD1. There was no downregulated DEP involved in malaria and African trypanosomiasis. To determine the signal transduction pathways and biochemical metabolic pathways of the 35 DEPs, KEGG pathway enrichment analysis was carried out. The results indicated that the DEPs were enriched with the terms malaria and African trypanosomiasis (Figure S5).

### 2.6. Eukaryotic Orthologous Groups (KOG) Functional Analysis of DEPs

KOG functional analysis can predict the possible functions of proteins and generate functional classification statistics by using the KOG database, a database for the orthologous classification of eukaryotic proteins. The KOG functional analysis results of the DEPs are shown in Figure S6. The results indicated that most of the DEPs were clustered in the functions of posttranslational modification, protein turnover, chaperones, signal transduction mechanisms, cytoskeleton, carbohydrate transport, and metabolism. 

### 2.7. Protein–Protein Interaction (PPI) Network Analysis

PPI network analyses of the DEPs were obtained with the STRING 11.0 database, using a confidence score higher than 0.9 (Figure S7). Among all DEPs, the top two hub proteins with the highest node degrees (that is, with the most neighbors) were matched as ALDOA and ORM2. These results suggest that these two proteins may contribute to the occurrence or development of SAP. 

### 2.8. The Plasma Concentration of These Biomarkers of Patients with AP

**Figure 5:**
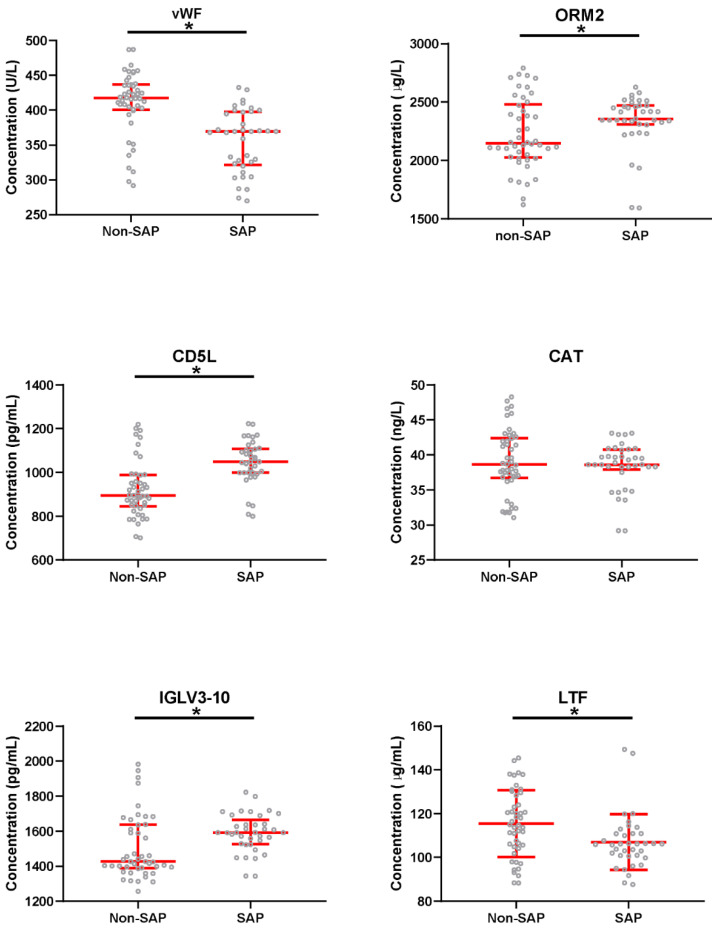
Comparison of marker levels in patients with severe acute pancreatitis or non-severe acute pancreatitis. VWF, von Willebrand factor; ORM2, alpha-1-acid glycoprotein 2; CD5L, CD5 antigen-like; CAT, catalase; IGLV3-10, immunoglobulin lambda variable 3–10; LTF, lactotransferrin; SAP, severe acute pancreatitis. * *p* < 0.05 (significant difference).

From the perspective of FC, *p* value, and clinical significance, 6 proteins were selected from the above 35 DEPs as biomarkers to predict the severity of AP. To validate the plasma concentration of these candidate biomarkers, we enrolled 83 patients with AP, comprised 37 patients with SAP (SAP group) and 46 patients with non-SAP (non-SAP group) (Table S3), and analyzed the levels of such candidates in these 83 patients using commercial ELISA kits. Table 3 below shows plasma concentration of these biomarkers in 83 patients with AP, in which, compared with non-SAP, there was no significant discrepancy in the SAP group, regarding CAT. In contrast, the VWF, ORM2, CD5L, IGLV3-10, and LTF showed significant discrepancies between the two groups (Figure 5).

### 2.9. Prediction Performance of These Biomarkers

**Figure 6:**
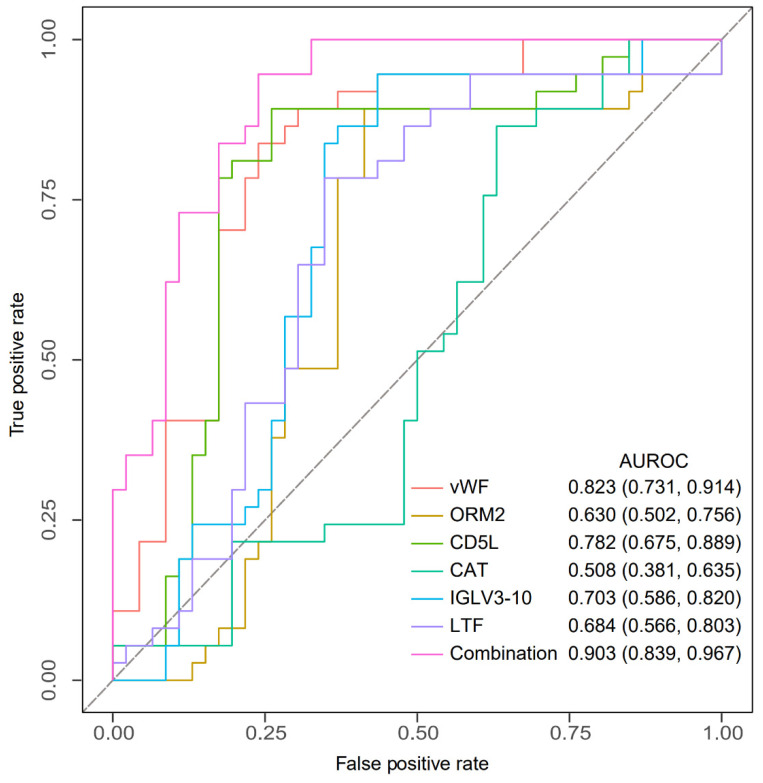
The receiver operating characteristic curves and area under the receiver operating characteristic curves of these candidate biomarkers. VWF, von Willebrand factor; ORM2, alpha-1-acid glycoprotein 2; CD5L, CD5 antigen-like; CAT, catalase; IGLV3-10, immunoglobulin lambda variable 3-10; LTF, lactotransferrin; AUROC, the area under the receiver operating characteristic; combination, a combination of these six candidate biomarkers; conbination-1, a new combination that catalase was removed from the above six candidate biomarkers.

To validate the prediction performance of these candidate biomarkers, the ROC curves were conducted (Table 3, Figure 6). The area under the receiver operating characteristic (AUROC) of 0.903 (95% CI: 0.839, 0.967) indicated that this combination of these six candidate biomarkers had good accuracy for predicting the severity of AP. Its specificity, sensitivity, negative predictive value, and positive predictive value were 0.761 (95% CI: 0.612, 0.874), 0.946 (95% CI: 0.818, 0.993), 0.946 (95% CI: 0.818, 0.974), and 0.761 (95% CI: 0.612, 0.965), respectively. Since there was no significant difference in plasma levels of CAT between the two groups, when it was removed from the combination (combination-1), the AUROC of 0.880 (95% CI: 0.806, 0.954) indicated that combination-1 also had good accuracy for predicting the severity of AP. Its specificity, sensitivity, negative predictive value, and positive predictive value were 0.848 (95% CI: 0.711, 0.937), 0.892 (95% CI: 0.746, 0.970), 0.907 (95% CI: 0.776, 0.963), and 0.825 (95% CI: 0.676, 0.948), respectively. Additionally, as shown in Figure 6, compared with the single index of VWF, ORM2, CD5L, CAT, IGLV3-10, or LTF levels, the prediction performance of this combination and combination-1 were also significantly higher.

## 3. Discussion

SAP has a high fatality rate, with effective treatment limited because its pathogenesis is still unclear. Proteomics may detect a large number of protein expression level changes in patients with SAP, which may prove helpful for quickly discovering clues in the occurrence and development of the disorder. For example, a recent proteomic study assessing pancreatitis in patients with alcoholic AP detected significant changes in plasma protein expression levels [11]. Another proteomic study found that the expression of PRSS1 in pancreatic acinus of mice with chronic pancreatitis was also significantly altered [12]. The present study used the DIA method in quantitative proteomics to analyze the expression levels of plasma proteins in patients with SAP and healthy controls. Our results indicated that, compared with the control group, there were 35 DEPs, with the expression levels of 6 proteins significantly increased and 29 proteins significantly decreased. In addition, 164 DEPs were detected only in the experimental group. Subsequent KEGG signaling pathway analysis showed that metabolic pathways were significantly changed in patients with SAP. These results provide valuable information for understanding the pathogenesis of SAP. Moreover, 6 proteins selected from 35 DEPs were successfully quantified. After statistical analysis, the AUROC and sensitivity of the combination of these six candidate biomarkers—VWF, ORM2, CD5L, CAT, IGLV3-10, and LTF—were 0.903 (95% CI: 0.839, 0.967) and 0.946 (95% CI: 0.818, 0.993), respectively. Ultimately, these six proteins were matched as candidate biomarkers of the disease severity of AP.

In the early stage of AP, inflammation occurs in pancreatic tissues, which secrete various inflammatory factors and recruit anti-inflammatory cells (such as neutrophils and macrophages) to local tissues for damage repair. The results of the present study indicated that the levels of TF, CD5L, and LTF, the proteins involved in the inflammatory response, were decreased in plasma. Among them, TF, an *N*-glycosylated glycoprotein in the acute stage of AP, has been shown to be at a lower level during sepsis [13]; therefore, it was speculated that changes in TF could reflect the intensity of the inflammatory response [14]. CD5L, also known as Sp alpha, is a soluble protein that belongs to the B group of the scavenger receptor-rich cysteine superfamily secreted by macrophages and has been shown to promote the survival of macrophages [15,16]. LTF is an iron-binding protein that is present in specific granules of neutrophils, and LTF levels are positively correlated with neutrophil activation [17]. The level of LTF is closely related to the level of neutrophils in human joint fluid, which may reflect the degree of the inflammatory response [18]. The changes in the expression levels of these proteins in SAP may be related to the course of disease compensation. However, the CAT and RBP4 proteins are involved in oxidative stress, and they showed significant expression level changes in the plasma of patients with SAP in the present study, with the CAT levels significantly increased and RBP4 levels significantly decreased. Oxidative stress is the main pathogenic factor in AP. CAT reduces the production of reactive oxygen species to remove hydrogen peroxide and inhibit pancreatic inflammation [19]. By contrast, RBP4 stimulates the production of oxygen-active free radicals that damage cells [20]. Thus, changes in these proteins during SAP may suggest the compensatory inhibition of the body, in response to the increased oxidative response. In addition, our results also showed that the plasma expression levels of proteins involved in immune regulation, including IGKV4-1, IGHG3, IGLV1-51, IGKV1-16, IGKV3D-15, IGKV1-39, IGKV1-17, IGLV3-10, and IGKV1D-16, were also significantly reduced. Recent studies have shown that CD4+ T cell-related immune responses may be one of the causes of the development and progression of pancreatitis [21]. Some innate immune cell levels are increased significantly during the acute phase (16 h) and then decreased to baseline during the recovery phase (30 h to 7 days), whereas adaptive immune cell levels decrease during the acute phase and then recover to baseline [22]. However, patients with SAP may develop immunosuppression, resulting in pancreatic necrosis and secondary infection [23,24]. IGKV4-1 is involved in the protection of acute rejection and considered an effective biomarker for the early identification of mantle cell lymphoma [25,26]. IGHG3, which is also involved in acute rejection [27], may contribute to platelet activation during ischemic stroke, and it is expected to be a potential target for the treatment of the early phase of stroke [28]. Studies from other groups have found that IGHM is abnormally expressed in patients with conjunctivitis-related systemic lupus erythematosus and cholangiocarcinoma [29,30]. In our study, several of the proteins involved in immune regulation were significantly reduced in plasma, indicating considerable immunosuppression in patients with SAP and suggesting that these proteins may be potential biomarkers for SAP.

The dysfunction of small vascular endothelial cells and abnormal coagulation function are also important factors in the development of acute pancreatitis. Our results showed that the expression levels of the five proteins involved in the regulation of endothelial cell function were significantly altered in the plasma of SAP patients, including significantly increased HbA1 and significantly decreased FBLN1, ECM1, RBP4, and DST. HbA1 expressed by endothelial cells captures nitric oxide, thereby preventing the diffusion of nitric oxide from endothelial cells to vascular smooth muscle and promoting vasoconstriction to control blood pressure and tissue perfusion [31]. FBLN1, an intercellular component of connective tissue, plays an important role in inducing angiogenesis and promoting the structural integrity of vascular walls [32,33]. In addition, previous studies have shown that ECM1 may interact with FBLN1 to participate in the composition of basement membrane proteins, and it may be critical for promoting angiogenesis [34]. Studies have indicated that RBP4 and DST may play a role in regulating endothelial barrier function and integrity in the body [35]. Therefore, changes in the expression levels of these proteins in patients with SAP may be closely related to the development of pancreatitis. In addition, the expression levels of three proteins involved in the regulation of blood clotting, FBLN1, IGHG3, and VWF, were also significantly reduced in the plasma of patients with SAP. FBLN1 is considered to be an extracellular matrix glycoprotein. In addition to participating in the regulation of vascular function, FBLN1 binds to fibrinogen, mediates platelet adhesion, and is a component of the newly formed fibrin-containing thrombus [36]. VWF is a large molecular glycoprotein produced by endothelial cells. A deficiency of VWF will lead to platelet adhesion, aggregation disorder, and decreased coagulation factor VIII procoagulant activity, leading to a bleeding tendency [37]. The present study found that the expression of VWF in plasma was significantly lower in patients with SAP, suggesting that decreased VWF may be a mechanism underlying the bleeding tendency in SAP patients. The rare complication of thrombotic thrombocytopenic purpura with AP has also been reported clinically, and it is speculated that the systemic inflammatory reaction may affect the activity of VWF, but there is no experimental evidence to confirm this [11,38]. However, Waldron et al. [11] performed a proteomic analysis of plasma samples from patients with alcoholic AP and found that, compared with the control group, the VWF levels in these patients were significantly higher. This is contrary to the results of the present study. We believe that the discrepant findings may be caused by different pathogeneses of different types of AP. Our findings suggest that changes in these related proteins may affect the occurrence, development, and prognosis of SAP by affecting endothelial cell functions and coagulation. However, our findings and this speculation need to be confirmed by experimental studies.

Our KEGG pathway enrichment analysis of the DEPs found that the metabolic pathway was the signal transduction pathway with the highest degree of enrichment. Metabolic pathways regulate the decomposition (catabolism) or synthesis (anabolism) of small molecules in cells or tissues by triggering a series of enzyme-mediated biochemical reactions. For example, during aerobic respiration, the synthesis of many key cellular molecular precursors is regulated by glycolysis and the Krebs cycle metabolic signaling pathways. Patients with SAP often display changes in blood glucose and lipid levels, and severe pancreatic injury may lead to diabetes and other complications. Therefore, we speculate that metabolic pathways may greatly contribute to the occurrence and development of SAP. Our results showed that 10 DEPs, namely BTD, FBLN1, AHSG, FETUB, GPLD1, TF, RBP4, CD5L, PCYOX1, and IGFALS, play important roles in substance metabolism. Biotin is a carboxylated coenzyme that is essential in the synthesis of glucose, fatty acids, and branched-chain amino acids [39], and BTD has a crucial role in the synthesis of biotin. Therefore, when BTD is deficient, the metabolic abnormalities associated with these three critical substances, such as organic aciduria, hyperammonemia, and secondary ketoacidosis, will occur [40]. FBLN1 is abundant in the plasma and arterial walls of patients with type 2 diabetes and can be used as a predictor of cardiovascular mortality in patients with type 2 diabetes [41]. An independent risk factor for type 2 diabetes is the plasma levels of AHSG, which is an insulin receptor inhibitor secreted by the liver and, along with FETUB [42,43], thought to be an important factor in insulin resistance in obesity [44,45]. GPLD1 serves as a biomarker that effectively differentiates latent autoimmune diabetes in adults from type 2 diabetes in the early stage [46], and it was shown that the circulating GPLD1 concentration in diabetic rats could be restored to normal levels by exercise training [47]. Masi et al. [48] found that TF may have predictive value for the occurrence of long-term complications in patients with diabetes, but the specific mechanisms remain to be studied. It has also been reported that RBP4 secreted by the liver and adipose tissue may be involved in insulin resistance, diabetes, and coronary artery disease [49,50]. Jugnam-Ang et al. [51] used proteomics to show abnormal expression of RBP4 and CD5L in the plasma of patients with hyperlipidemia. They believed that CD5L may inhibit the secretion of RBP4 from adipose tissue through inflammatory and metabolic processes. PCYOX1 is mainly expressed in the liver and interacts with a variety of proteins, thus playing an important role in substance metabolism [52]. Through proteomic analysis, Mavreli et al. [53] found that PCYOX1, ECM1, HSPG2, CNDP1, and TSP-4 proteins have great potential in the early prediction of gestational diabetes mellitus and can serve as biomarkers for the prenatal screening of the disorder. IGFALS is also a plasma protein synthesized by the liver, and it improves glucose tolerance and insulin resistance by prolonging the half-life and increasing the circulating concentration of insulin-like growth factor-1 [54,55]. These metabolism-related DEPs found in the present study suggest that the occurrence and development of SAP are closely related to metabolic disorders. However, further experimental studies are needed to confirm our hypothesis. In addition, although this study found some DEPs through rigorous methods, there were still some limitations. The sample size of the control group was small. Secondly, the suboptimal sample size may increase the bias of some parameters, such as gender. Therefore, this study may provide a clue about SAP and can be improved by increasing the sample size in the future.

In summary, screening using DIA-based proteomic analysis detected some important DEPs in the plasma of patients with SAP. These DEPs are associated with the inflammatory response, oxidative stress, immunosuppression, impaired endothelial cell function, abnormal blood clotting, and substance metabolism disorders. Moreover, 6 candidate biomarkers—VWF, ORM2, CD5L, CAT, IGLV3-10, and LTF—selected from 35 DEPs were matched as the candidate biomarkers of disease severity of AP. They may also be potential biomarkers for the clinical diagnosis of SAP, provide potential mechanisms for the occurrence and development of SAP, and inform future research on the prevention and treatment of SAP.

## 4. Materials and Methods

### 4.1. Experimental Design

A flowchart of the experimental design is shown in Figure S8.

### 4.2. Sample Collection

Patients with AP who were admitted early to the Department of Emergency of the Second Affiliated Hospital of Anhui Medical University from May 2020 to April 2021 were enrolled in this retrospective study. Patients who met two of the following three criteria were regarded as having AP: (1) typical findings from computed tomography or abdominal ultrasonography; (2) characteristic symptoms with continuous abdominal pain; and (3) serum lipase or amylase levels that were higher than three times the normal reference upper limit. According to the Atlanta classification criteria, revised in 2012, SAP was defined as AP accompanied by continuous single or multiple organ dysfunction lasting at least 48 h [56]; if not, it is defined as non-SAP. Patients with AP who met any of the following conditions were excluded: (1) age older than 65 years or younger than 18 years; (2) pancreatitis induced by trauma; (3) anemia; (4) chronic pancreatitis; (5) malignant tumors; (6) pregnancy; (7) time from onset of abdominal pain to hospital admission ≥48 h; and (8) unavailable medical records. Healthy volunteers undergoing medical interview and physical examination were enrolled as the control group. Plasma samples from all individuals were collected within 24 h after admission. The study was executed according to the principles of the Declaration of Helsinki, and written informed consent was obtained from the study participants prior to study commencement. The study protocol was approved by the Medical Ethics Committee of the Second Affiliated Hospital of Anhui Medical University (approval number YX2021-062).

### 4.3. Extraction and Quality Control of Proteins 

A Pierce Top 12 Abundant Protein Depletion Spin Column (Thermo Fisher, Waltham, MA, USA, Cat. No. 85165) was used to remove high-abundance proteins from the plasma samples. The Bradford method was used to determine the depleted plasma protein concentration. The quality of the samples was evaluated by protein quantification and SDS-PAGE.

### 4.4. Protein Digestion 

After protein quantification, 60 μg of protein solution was placed in a centrifuge tube and mixed with dithiothreitol (5 μL of 1 mol/L) solution (Genview, Houston, TX, USA, Cat. No. CD116-25g). The reaction was incubated at 37 °C for 1 h. Iodoacetamide (20 μL of 1 mol/L) solution (Vetec, Singapore, Cat. No. V900335-5g) was added, and the components were mixed and kept in darkness at room temperature for 1 h. All samples were then pipetted into an ultrafiltration tube, and the collected solution was removed after centrifugation. Samples were diluted with 100 μL of urea (8 mol/L; Sigma, Darmstadt, Germany, Cat. No. U5378) and 100 mmol/L Tris-HCl (Amresco, Solon, OH, USA, Cat. No. 0497) and centrifuged at 14,000× *g* for 10 min in duplicate. After the collected solution was discarded, samples were diluted with 100 μL of 50 mmol/L NH_4_HCO_3_ (Sigma, Cat. No. 09830-500G) and centrifuged in triplicate. Trypsin buffer (Promega, Madison, WI, USA, Cat. No. V5113) was added, mixed, and then incubated for 12–16 h at 37 °C. The enzymatic hydrolysate was transferred to a new centrifuge tube.

### 4.5. Collection of Mass Spectral Data 

The samples were redissolved in 40 μL of 0.1% formic acid aqueous solution (Sigma, Cat. No. 94318) for liquid chromatography–tandem mass spectrometry analysis. The mobile phases consisted of phase A (2% acetonitrile/0.1% formic acid/98% water) and B (80% acetonitrile/0.08% formic acid/20% water). The samples flowed through the analytical column (Thermo Fisher Scientific, Acclaim PepMap RSLC 50 μm × 15 cm, nano viper, P/N164943) at a steady rate of 300 nL/min and were separated with a nonlinear increasing gradient. The gradient for phase B increased linearly at 0–5 min from 0% to 1%, at 5–95 min from 1% to 28%, at 95–110 min from 28% to 38%, at 110–115 min from 38% to 100%, and at 115–120 min remained at 100%. After separation by capillary high-performance liquid chromatography, each sample was analyzed using an Orbitrap Fusion Lumos mass spectrometer (Thermo Fisher Scientific).

### 4.6. Analyses of Raw Mass Spectral Data

Raw data from data-dependent acquisition were processed and analyzed by Spectronaut Pulsar X software (Biognosys AG), which was set up to search the Uniprot_HomoSapiens_20367_20200226 database (http://www.uniprot.org; accessed on 6 may 2022), assuming that trypsin was the digestion enzyme. Carbamidomethyl (C) was specified as the fixed modification. Acetyl (protein N-term) and oxidation (M) were specified as the variable modifications. The FDR cutoff for the precursor and peptide levels was 1%. Spectronaut Pulsar X software was also applied for DIA analysis with default settings, and the FDR was also set as 1%. 

A histogram of the frequency distribution of peptide length was used to evaluate whether the protease used in the study was reasonable. To intuitively compare the differences in protein expression levels between the two groups, a histogram of the frequency distribution of the ratios of the proteins found in each group (experimental vs. control) was used. Compared with the control group, proteins with FC ≤ 0.667 or ≥1.5 (*p* < 0.05) were considered to be DEPs. Correlation coefficient matrix creation, volcano plot construction, hierarchical clustering, heatmap construction, and statistical analyses were all conducted in R software.

### 4.7. Bioinformatics Analyses

To investigate the basic functional and metabolic characteristics, including cellular composition, biological process, and molecular function [57], these DEPs were assessed by GO analysis (http://geneontology.org/; accessed on 6 May 2022). The signaling pathways of these DEPs were enriched and analyzed by using KEGG pathway database (https://www.kegg.jp/; accessed on 6 May 2022). All DEPs were compared with the KOG database (https://mycocosm.jgi.doe.gov/help/kogbrowser.jsf; accessed on 6 May 2022) by protein BLAST (E-value < 10^−5^), and the corresponding KOG annotation results were obtained. PPI network analyses were applied to find the interactions among all DEPs by using the STRING database 11.0 (https://string-db.org/; accessed on 6 May 2022).

### 4.8. Elisa

Analyses of samples were performed using commercially available ELISA kits, in accordance with the manufacturer’s instructions. The following ELISA kits were used: VWF, ORM2, CD5L, CAT, IGLV3-10, and LTF (Shanghai Enzyme-linked Biotechnology Co., Ltd., Shanghai, China). Briefly, diluted serum was added to each well and incubated according to the manufacturer’s instructions. Upon completion of the assay procedure, the plate was read at 450 nm using a microplate reader (BioTek, Winooski, VT, USA).

### 4.9. Statistical Analysis

In this study, Shapiro–Wilk test was used to confirm whether the data conform to a normal distribution. Measurement data are described as mean ± standard deviation or median (interquartile ranges), and counting data are presented as percentages (%), using Mann–Whitney U-test or unpaired Student’s test, when appropriate, to compare these continuous variables. Meanwhile, the chi-squared or Fisher exact tests were used to compare the categorical variables. By the way, to evaluate the prediction performance of the clinical application of these indexes, the ROC curve was conducted. *p* values < 0.05 were considered statistically significant. GraphPad Prism (version, 8.0.1; GraphPad Software, San Diego, CA, USA), SPSS (version, 23.0; SPSS, Chicago, IL, USA), and R (version, 4.0.3; Illinois Institute of Technology, Chicago, IL, USA) software were used to perform statistical analyses.

## 5. Conclusions

In the present study, the results were obtained using 13 plasma protein samples collected from patients with SAP and healthy controls, and they were analyzed using DIA-based proteomics, which indicated that the pathogenesis of SAP involves multiple proteins and important signaling pathways, including metabolic pathways. Moreover, six candidate biomarkers—VWF, ORM2, CD5L, CAT, IGLV3-10, and LTF—were matched as candidate biomarkers of disease severity of AP. These candidate biomarkers may be involved in processes associated with AP, such as coagulation, immunity, and inflammation. Therefore, our findings provide specific proteins that may be useful in the diagnosis and prognosis of SAP and may offer not only new underlying theoretical bases for the occurrence and development of SAP but also potential therapeutic strategies.

## Figures and Tables

**Table 1:** Baseline characteristics of patients with severe acute pancreatitis and healthy controls.

Features/Groups	Severe Acute Pancreatitis (*n* = 10)	Healthy Controls (*n* = 3)	Statistical Result	*p* Value
Sex, *n* (%)				1.00 ^a^
Male	4 (40)	1 (33.33)		
Female	6 (60)	2 (66.66)		
Age (years)	51.10 ± 14.44	32.67 ± 5.13	*t* = 2.114	0.058 ^b^
Smoker (%)	3 (30)	1 (33.33)		1.00 ^a^
Alcoholism (%)	2 (20)	1 (33.33)		1.00 ^a^
BMI (kg/m^2^)	22.44 ± 4.57	22.30 ± 0.50	*t* = 0.095	0.926 ^b^
SBP (mmHg)	133.20 ± 13.93	119.33 ± 4.51	*t* = 1.652	0.127 ^b^
DBP (mmHg)	86.50 ± 9.64	76.00 ± 5.00	*t* = 1.777	0.103 ^b^
FBG (mmol/L)	9.47 ± 2.25	4.65 ± 0.75	*t* = 3.545	0.005 ^b^
TC (mmol/L)	5.46 ± 1.65	5.74 ± 0.20	*t* = −0.278	0.786 ^b^
TG (mmol/L)	4.52 ± 2.88	2.09 ± 0.58	*t* = 1.412	0.186 ^b^
WBC (×10^9^/L)	15.97 ± 5.66	5.93 ± 1.48	*t* = 2.956	0.013 ^b^
Amylase (U/L)	1490.40 ± 873.33	41.33 ± 5.03	*t* = 5.247	0.001 ^b^

BMI, body mass index; SBP, systolic blood pressure; DBP, diastolic blood pressure; FBG, fasting blood glucose; TC, total cholesterol; TG, triglycerides; WBC, white blood cell. The *p* value was obtained for comparison of the groups with following tests: ^a^ Fisher exact test; ^b^ unpaired Student’s *t*-test.

**Table 2:** Thirty-five differentially expressed proteins between the experimental and control groups.

Regulation	Protein Accession Identification	Gene Name	Protein Description	FC	*p* Value
UP	P04040	CAT	Catalase	22.61	0.020
UP	P00915	CA1	Carbonic anhydrase 1	19.78	0.027
UP	P68871	HBB	Hemoglobin subunit beta	11.33	0.007
UP	P69905	HBA1	Hemoglobin subunit alpha	10.46	0.017
UP	P69891	HBG1	Hemoglobin subunit gamma-1	4.27	0.033
UP	P19652	ORM2	Alpha-1-acid glycoprotein 2	2.27	0.032
DOWN	P23142	FBLN1	Fibulin-1	0.66	0.004
DOWN	P02753	RBP4	Retinol-binding protein 4	0.66	0.009
DOWN	P02787	TF	Serotransferrin	0.65	0.012
DOWN	P43251	BTD	Biotinidase	0.64	0.010
DOWN	P06312	IGKV4-1	Immunoglobulin kappa variable 4-1	0.62	0.043
DOWN	P02765	AHSG	Alpha-2-HS-glycoprotein	0.60	0.008
DOWN	P01871	IGHM	Immunoglobulin heavy constant mu	0.60	0.025
DOWN	Q9UHG3	PCYOX1	Prenylcysteine oxidase 1	0.59	0.048
DOWN	Q9UGM5	FETUB	Fetuin-B	0.57	0.012
DOWN	P51884	LUM	Lumican	0.56	0.001
DOWN	P07477	PRSS1	Trypsin-1	0.56	0.004
DOWN	P0DOX8	-	Immunoglobulin lambda-1 light chain	0.56	0.007
DOWN	P80108	GPLD1	Phosphatidylinositol-glycan-specific phospholipase D	0.55	0.033
DOWN	P35858	IGFALS	Insulin-like growth factor-binding protein complex acid labile subunit	0.51	0.007
DOWN	P01860	IGHG3	Immunoglobulin heavy constant gamma 3	0.51	0.024
DOWN	P01701	IGLV1-51	Immunoglobulin lambda variable 1-51	0.47	0.027
DOWN	Q16610	ECM1	Extracellular matrix protein 1	0.45	0.027
DOWN	O43866	CD5L	CD5 antigen-like	0.45	0.000
DOWN	P04430	IGKV1-16	Immunoglobulin kappa variable 1-16	0.43	0.007
DOWN	A0A087WSY6	IGKV3D-15	Immunoglobulin kappa variable 3D-15	0.43	0.021
DOWN	P01597; P04432	IGKV1-39; IGKV1D-39	Immunoglobulin kappa variable 1-39; immunoglobulin kappa variable 1D-39	0.40	0.006
DOWN	P01599	IGKV1-17	Immunoglobulin kappa variable 1-17	0.39	0.023
DOWN	A0A075B6K4	IGLV3-10	Immunoglobulin lambda variable 3-10	0.38	0.000
DOWN	P02788	LTF	Lactotransferrin	0.33	0.001
DOWN	Q9H251	CDH23	Cadherin-23	0.24	0.024
DOWN	Q03001	DST	Dystonin	0.18	0.047
DOWN	P04275	VWF	Von Willebrand factor	0.16	0.000
DOWN	P01601	IGKV1D-16	Immunoglobulin kappa variable 1D-16	0.15	0.018
DOWN	P12883; P13533	MYH7; MYH6	Myosin-7; myosin-6	0.14	0.028

FC represents fold change, that is, the linear value of the quantitative ratio in the protein concentration between the experimental and control groups.

**Table 3:** Marker levels in patients with severe acute pancreatitis or non-severe acute pancreatitis.

Biomarkers	Non-SAP (*n* = 46)	SAP (*n* = 37)	Statistical Result	*p* Value	AUROC (95% CI)	COP	SEN (%)	SPE (%)
VWF (U/L)	417.38 (400.71–437.01)	369.43 (321.48–397.70)	*z* = −5.030	<0.001	0.823 (0.731,0.914)	400.71	76.09	83.78
ORM2 (µg/L)	2147.26 (2026.05–2480.25)	2357.29 (2308.36–2470.00)	*z* = −2.016	0.044	0.630 (0.502,0.756)	2206.79	89.19	58.70
CD5L (pg/mL)	894.24 (845.30–988.33)	1049.10 (998.81–1108.64)	*z* = −4.398	<0.001	0.782 (0.675,0.889)	962.14	89.19	73.91
CAT (ng/L)	38.62 (36.72–42.43)	38.62 (37.91–40.75)	*z* = −0.119	0.905	0.508 (0.381,0.635)	41.13	36.96	86.49
IGLV3-10 (pg/mL)	1426.93 (1389.85–1637.12)	1591.96 (1526.25–1664.96)	*z* = −3.170	0.002	0.703 (0.586,0.820)	1442.83	94.59	56.52
LTF (µg/mL)	114.83 (104.67–128.61)	106.18 (100.72–110.90)	*z* = −2.877	0.004	0.684 (0.566,0.803)	110.94	65.22	78.38

VWF, von Willebrand factor; ORM2, alpha-1-acid glycoprotein 2; CD5L, CD5 antigen-like; CAT, catalase; IGLV3-10, immunoglobulin lambda variable 3–10; LTF, lactotransferrin; SAP, severe acute pancreatitis; AUROC, the area under the receiver operating characteristic; CI, confidence interval; COP, cutoff point; SEN, sensitivity; SPE, specificity.

## Data Availability

The data presented in this study are available upon request from the corresponding author.

## Supplementary Materials

The following supporting information can be downloaded at: https://www.mdpi.com/article/10.3390/molecules27123880/s1, Figure S1. Sodium dodecyl sulfate–polyacrylamide gel electrophoresis. In the image, M indicates the lane with the markers. SAP1–SAP10 are the lanes with plasma samples obtained from patients 1–10 in the experimental group; NC1–NC3 are the lanes with plasma samples obtained from healthy volunteers 1–3 in the control group. Figure S2. Partial least-squares analysis of proteomic data. SAP1–SAP10 are the plasma samples obtained from patients 1–10 in the experimental group; NC1–NC3 are the plasma samples obtained from healthy volunteers 1–3 in the control group. Figure S3. Proteomic analysis of data-independent, acquisition–based quantitative data. (A) Histogram of the frequency distribution of the lengths of all peptides. (B) Histogram of the frequency distribution of the ratio of all proteins in the experimental group vs. control group. (C) Correlation coefficient matrix among 13 samples. NC1–NC3 indicates healthy control group volunteers 1–3; SAP1–SAP10, experimental group patients 1–10. Figure S4. Gene ontology (GO) functional enrichment analysis of 35 DEPs. The size of the circle represents the number of DEPs in the GO term. The color of the circle indicates the *p* value for the degree of enrichment. Red represents a smaller *p* value and higher enrichment degree. The Fisher exact test *p* value is the *p* value of the enrichment test obtained using the Fisher exact test; and −log10 (*p* value) is the log conversion of the Fisher exact test *p* value. Figure S5. Kyoto Encyclopedia of Genes and Genomes (KEGG) pathway enrichment analysis of 35 DEPs. The size of the circle represents the number of differentially expressed proteins in the KEGG term. The color of the circle indicates the *p* value for the degree of enrichment. Red represents a smaller *p* value and higher enrichment degree. The Fisher exact test *p* value is the *p* value of the enrichment test obtained using the Fisher exact test; −log10 (*p* value) is the log conversion of the Fisher exact test *p* value. Figure S6. Eukaryotic orthologous groups (KOG) functional analysis of all differentially expressed proteins (DEPs) between the experimental and control groups. The abscissa is the functional class code from the KOG database. The descriptive information for the functional class code is shown on the right side of the figure. The ordinate is the frequency of each functional class code. Figure S7. Protein–protein interaction (PPI) network analysis. PPI network analysis of all differentially expressed proteins (DEPs). Colored nodes represent query proteins and the first shell of interactors; white nodes represent the second shell of interactors; empty nodes represent proteins of unknown three-dimensional structure; filled nodes indicate that some three-dimensional structure is known or predicted. Edges represent protein–protein associations, and the associations are meant to be specific and meaningful; that is, the proteins jointly contribute to a shared function. This does not necessarily mean that they physically bind each other. Figure S8. Flowchart of the experimental design. GO: Gene ontology; KEGG: Kyoto Encyclopedia of Genes and Genomes; KOG: eukaryotic orthologous groups; PPI: protein–protein interaction; ROC: receiver operating characteristic. Table S1. Quantitative results of proteins detected in 13 samples. NC1–NC3 are control group patients 1–3; SAP1–SAP10 are experimental group patients 1–10. Table S2. Data for all proteins and peptides obtained by data-independent acquisition (DIA)-based proteomic analysis. All proteins and peptides represent all the proteins and peptides obtained by DIA-based proteomic analysis, respectively. Description of all protein list headers: AccessionID, the first protein ID given in the UniProt database; ProteinAccessions, number of the protein sequence given in the UniProt database; Qvalue, false discovery rate; Genes, gene name given in the UniProt database; ProteinDescriptions, functional description of proteins in the UniProt database based on the protein sequence; ProteinNames, protein name given in the UniProt database. Description of all peptide list headers: PEP.StrippedSequence, amino acid sequence of the peptide; PEP.IsProteinGroupSpecific, whether it is the only peptide segment (true or false); EG.PrecursorId, the ID number of precursors; PG.ProteinGroups, the proteome to which it belongs; PG.ProteinAccessions, login number of the protein to which it belongs; PG.Genes, gene name; PG.ProteinDescriptions, functional description of proteins in database, based on protein sequences; PG.ProteinNames, protein name. Table S3. Baseline characteristics of the remaining 83 patients with acute pancreatitis. BMI, body mass index; SBP, systolic blood pressure; DBP, diastolic blood pressure; TC, total cholesterol; TG, triglycerides; Glu, glucose; IL-1, interleukin-1; PCT, procalcitonin; WBC, white blood cell; SAP, severe acute pancreatitis. The *p* value was obtained for comparison of the groups with the following tests: ^a^ chi-square test; ^b^ unpaired Student’s *t*-test; ^c^ Mann-Whitney U test.

## Sample Availability

Not available.
