# Supplementary material for: DIA-Based Proteomic Analysis of Plasma Protein Profiles in Patients with Severe Acute Pancreatitis
Source: Molecules. 2022 Jun 17;27(12):3880. doi: 10.3390/molecules27123880 (PMC9230633; doi:10.3390/molecules27123880)
Supplement: Supplementary file 1 [file molecules-27-03880-s001.zip › Supplementary Files.pdf]

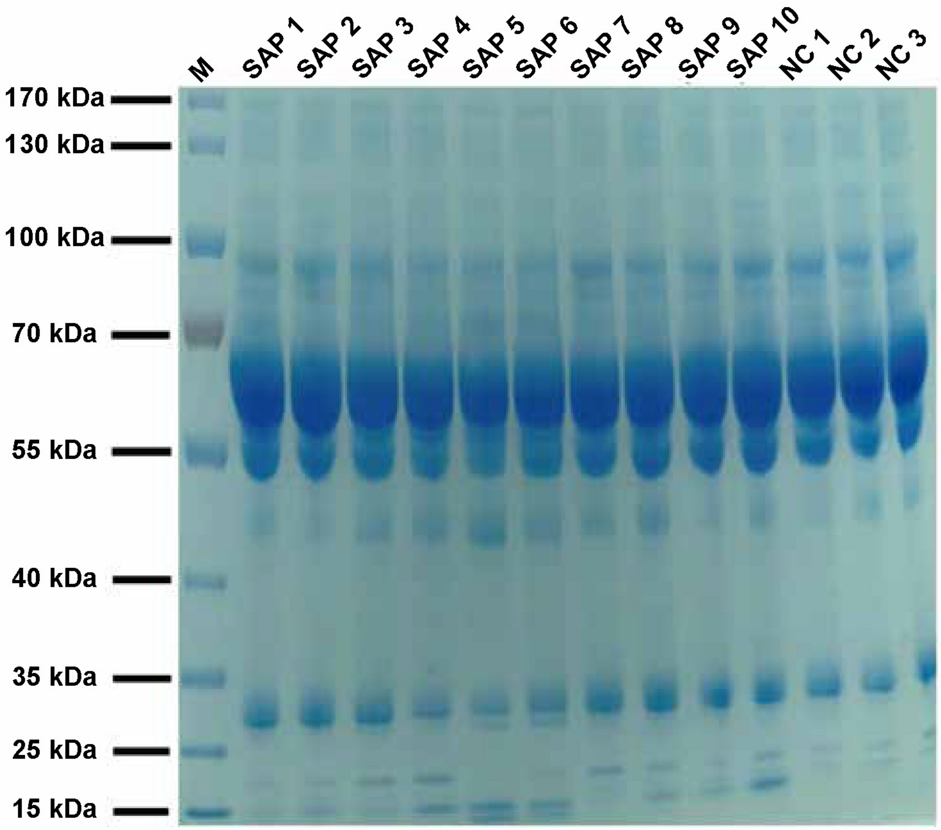

Figure S1. Sodium dodecyl sulfate–polyacrylamide gel electrophoresis. In the image, M indicates the lane with the markers. SAP1–SAP10 are lanes with plasma samples obtained from patients 1–10 in the experimental group; NC1–NC3 are lanes with plasma samples obtained from healthy volunteers 1–3 in the control group.

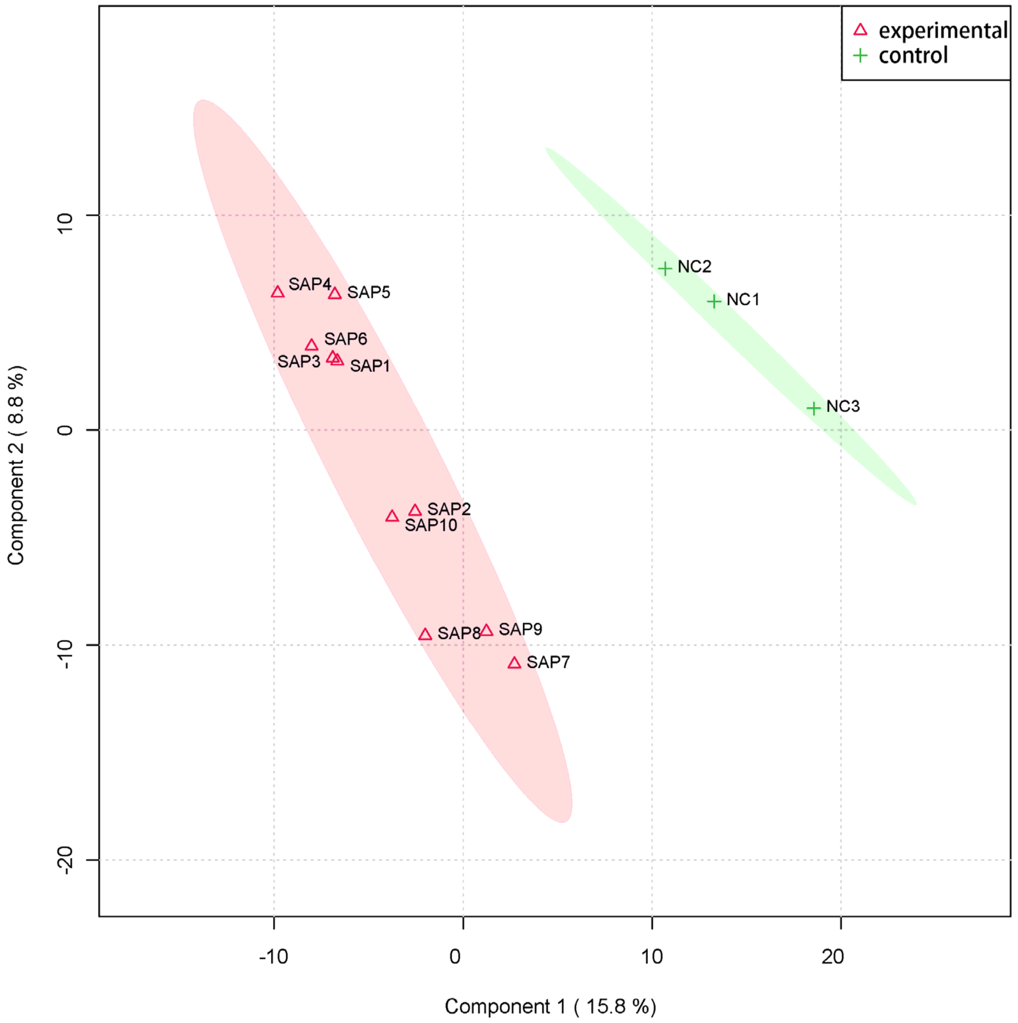

Figure S2. Partial Least-Squares analysis of proteomic data. SAP1–SAP10 are plasma samples obtained from patients 1–10 in the experimental group; NC1–NC3 are plasma samples obtained from healthy volunteers 1–3 in the control group.

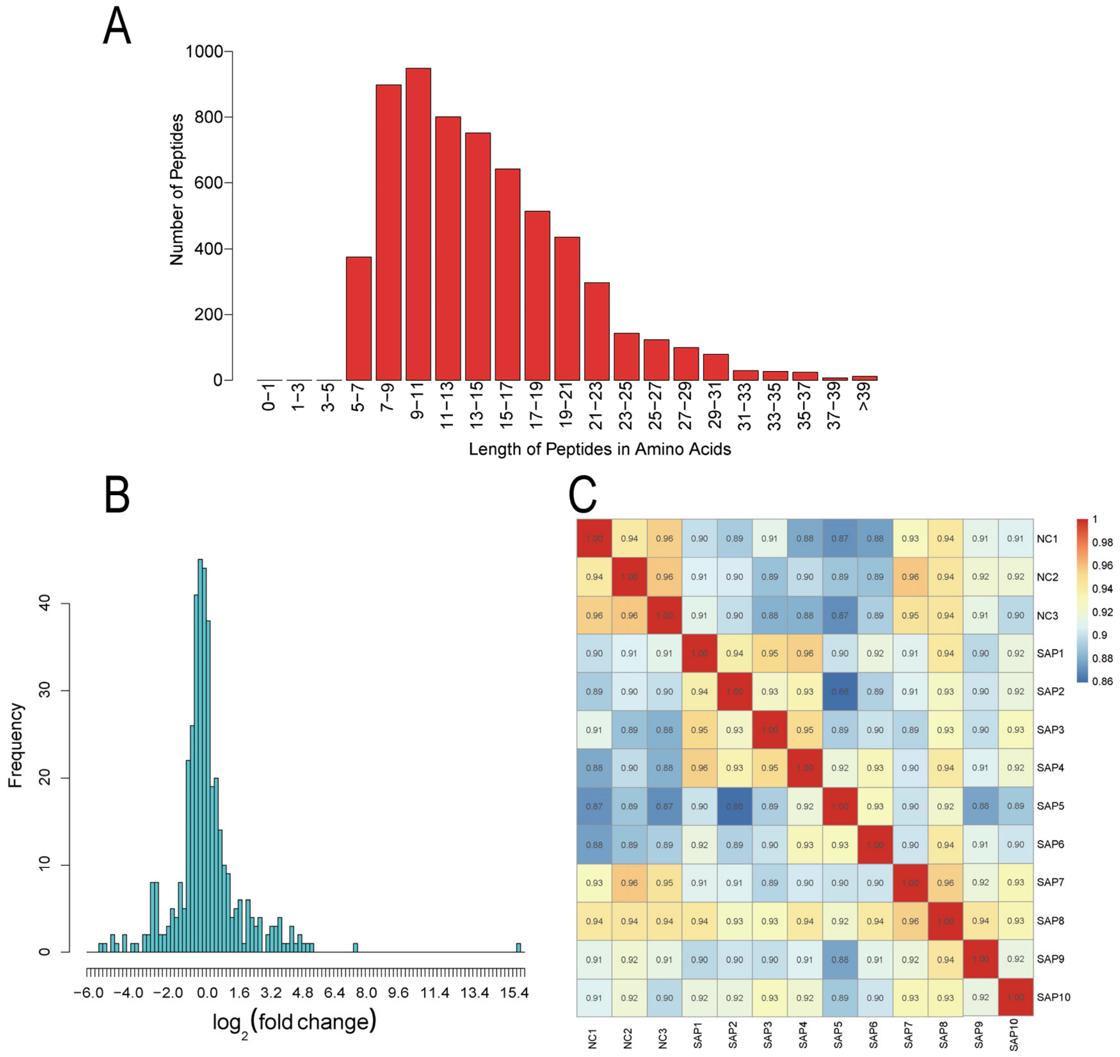

Figure S3. Proteomic analysis of data-independent acquisition–based quantitative data. (A) Histogram of the frequency distribution of the lengths of all peptides. (B) Histogram of the frequency distribution of the ratio of all proteins in the experimental vs. the control group. (C) Correlation coefficient matrix among 13 samples. NC1–NC3 indicates control group healthy volunteers 1–3; SAP1–SAP10, experimental group patients 1–10.

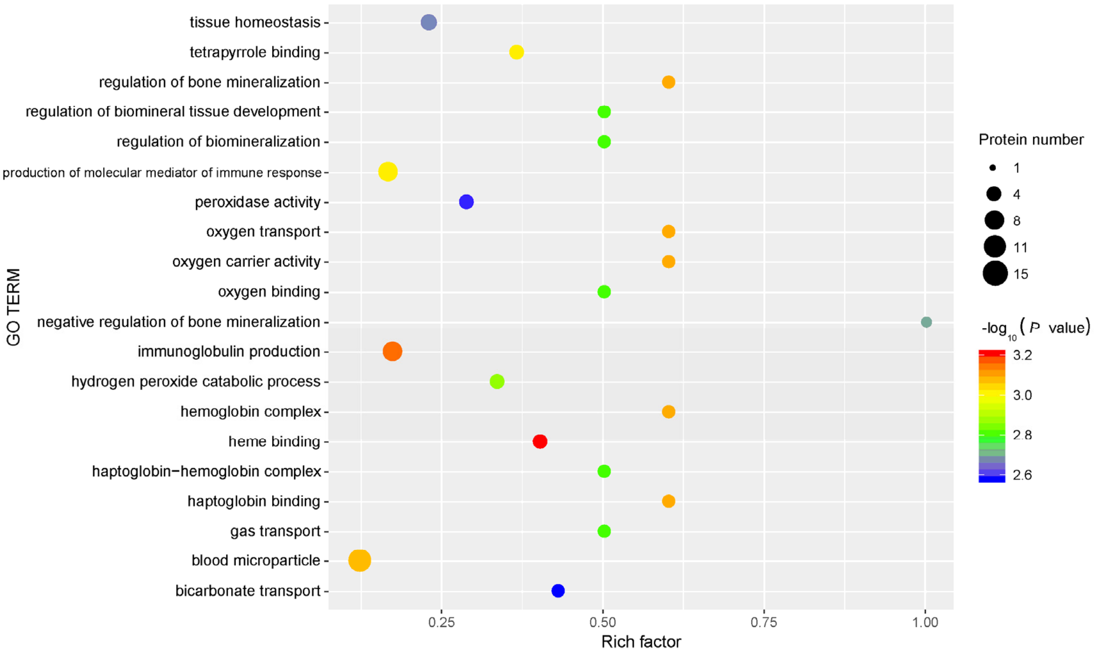

Figure S4. Gene Ontology (GO) functional enrichment analysis of 35 DEPs. The size of the circle represents the number of DEPs in the GO term. The color of the circle indicates the *P* value for the degree of enrichment. Red represents a smaller *P* value and a higher enrichment degree. The Fisher exact test *P* value is the *P* value of the enrichment test obtained using the Fisher exact test; and −log10(*P* value) is the log conversion of the Fisher exact test *P* value.

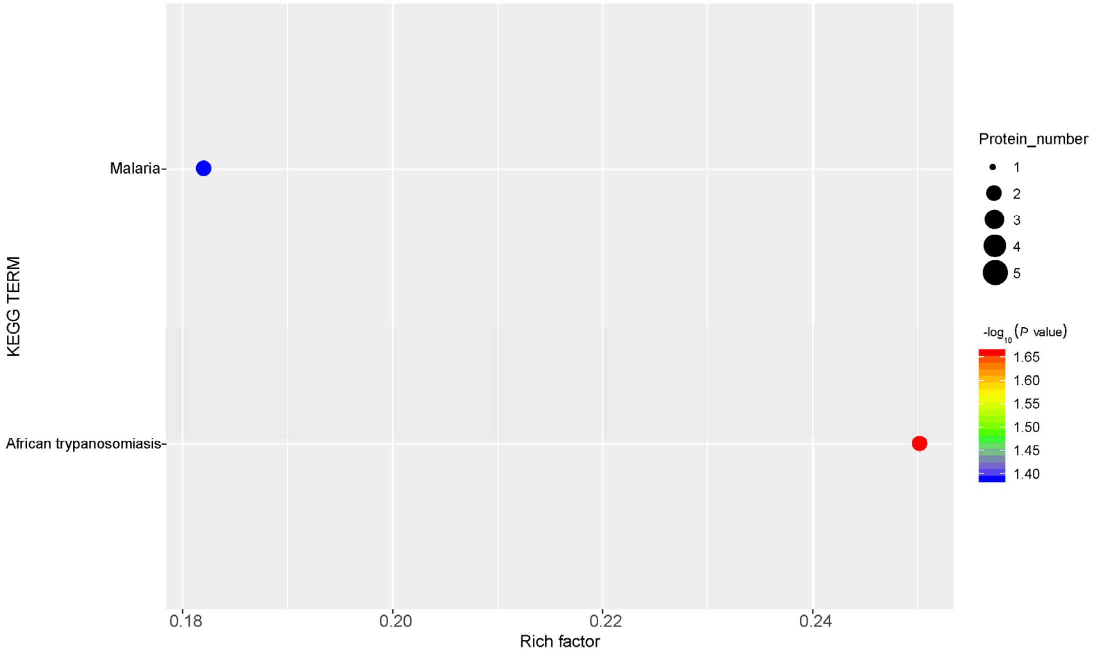

Figure S5. Kyoto Encyclopedia of Genes and Genomes (KEGG) pathway enrichment analysis of 35 DEPs. The size of the circle represents the number of differentially expressed proteins in the KEGG term. The color of the circle indicates the *P* value for the degree of enrichment. Red represents a smaller *P* value and a higher enrichment degree. The Fisher exact test *P* value is the *P* value of the enrichment test obtained using the Fisher exact test; and −log10(*P* value) is the log conversion of the Fisher exact test *P* value.

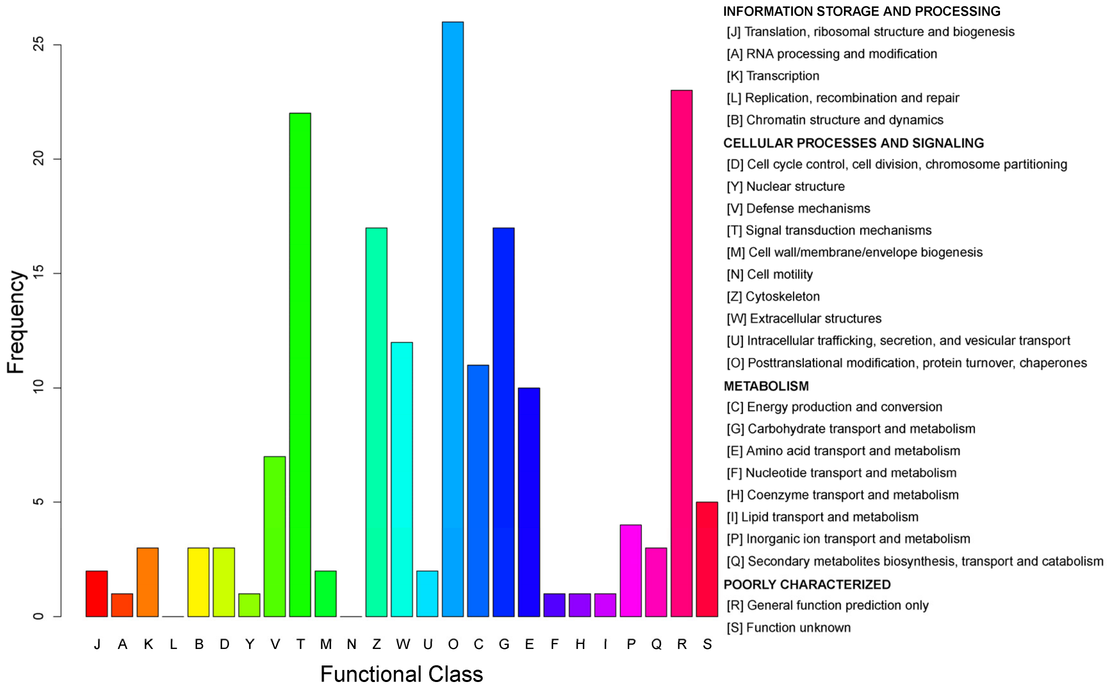

Figure S6. Eukaryotic Orthologous Groups (KOG) functional analysis of all differentially expressed proteins (DEPs) between the experimental and control groups. The abscissa is the functional class code from the KOG database. The descriptive information for the functional class code is shown on the right side of the figure. The ordinate is the frequency of each functional class code.

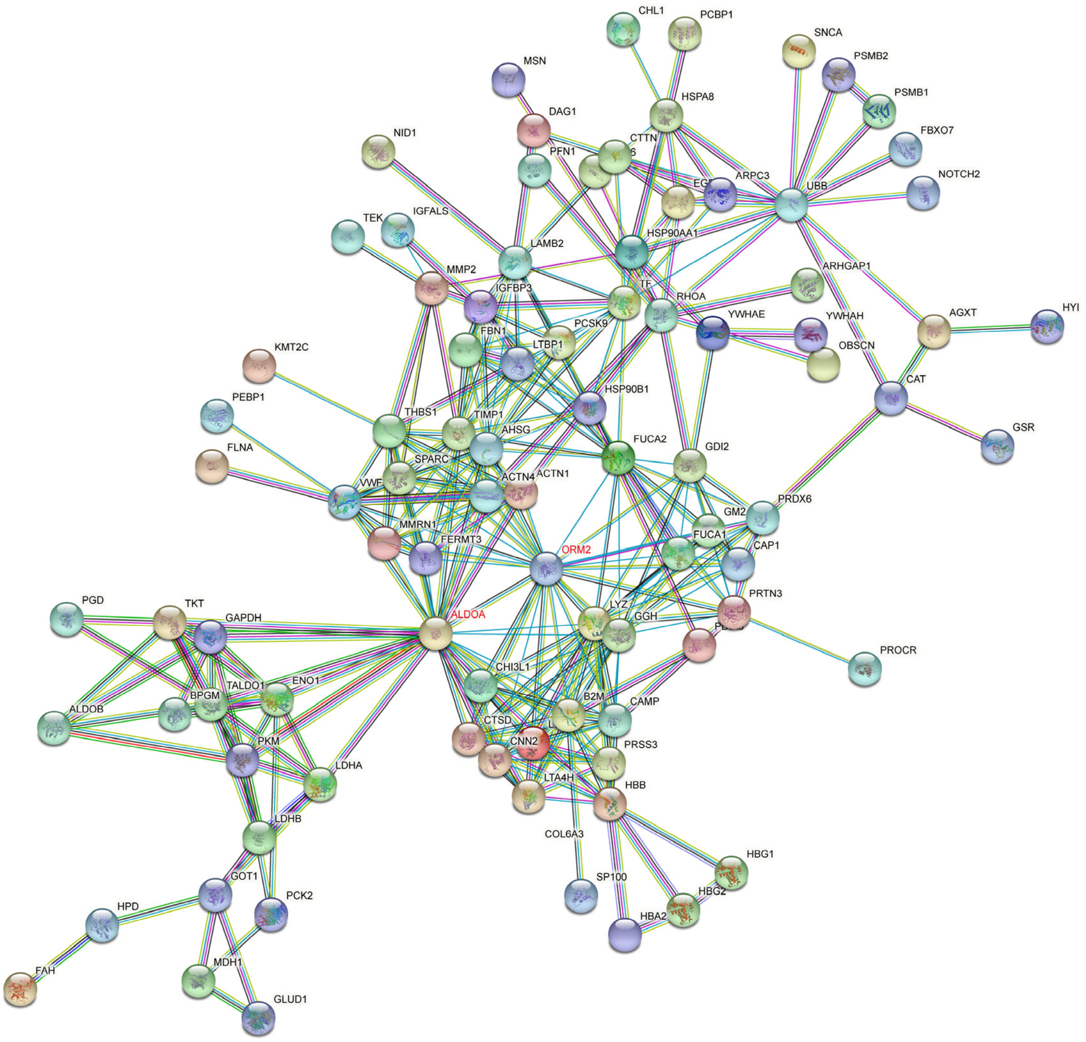

Figure S7. Protein-protein interaction (PPI) network analysis. PPI network analysis of all differentially expressed proteins (DEPs). Colored nodes represent query proteins and the first shell of interactors; white nodes represent the second shell of interactors; empty nodes represent proteins of unknown three-dimensional structure; filled nodes indicate that some three-dimensional structure is known or predicted. Edges represent protein-protein associations, and associations are meant to be specific and meaningful, that is, proteins jointly contribute to a shared function; this does not necessarily mean they physically bind each other.

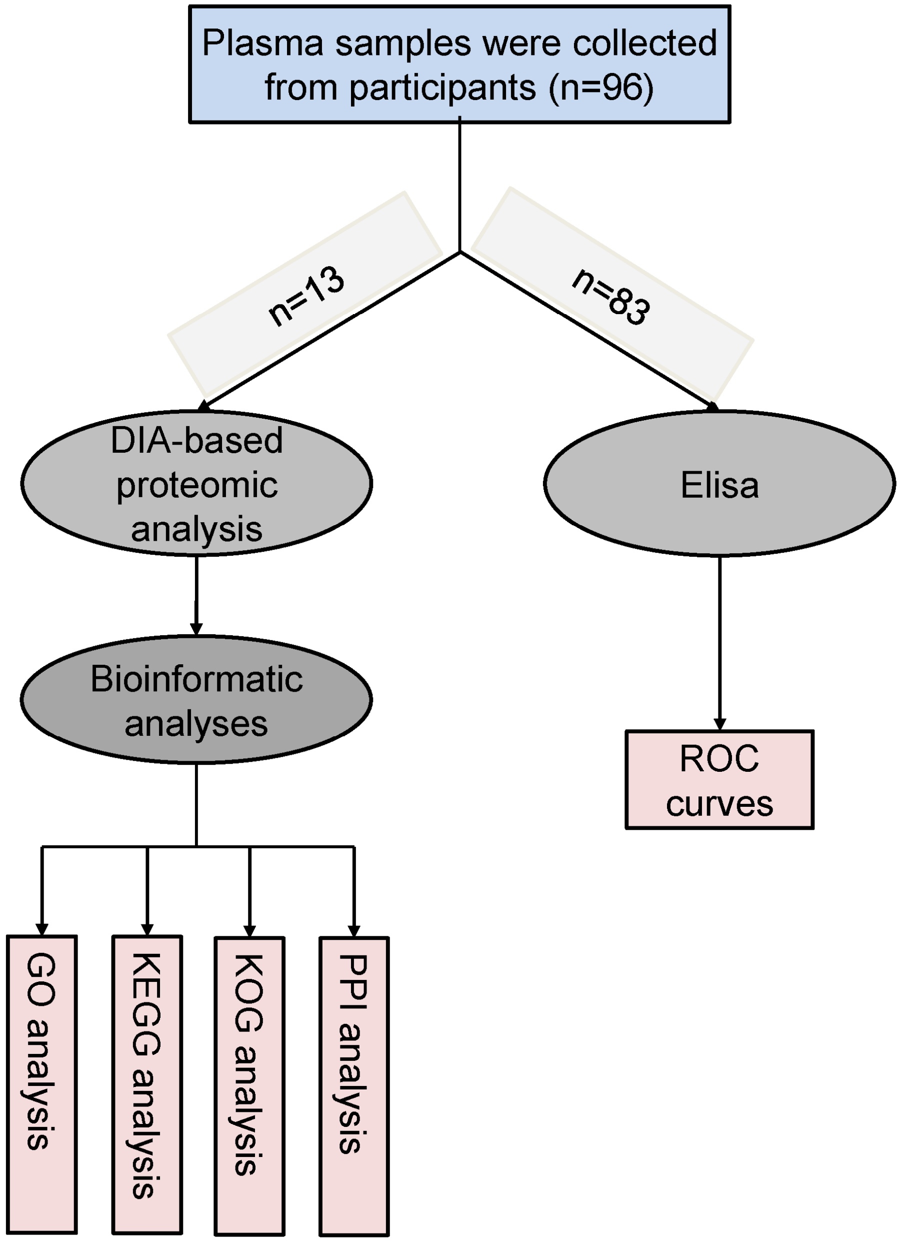

Figure S8. Flowchart of the experimental design. GO: Gene Ontology; KEGG: Kyoto Encyclopedia of Genes and Genomes; KOG: Eukaryotic Orthologous Groups; PPI: protein-protein interaction; ROC: receiver operating characteristic.

Table S1: Quantitative results of proteins detected in 13 samples

Sample identification	Protein concentration (μg/μL)	Total protein volume (μL)	Total protein (μg)
SAP1	19.1	480	9168
SAP2	18.6	480	8928
SAP3	15.9	480	7632
SAP4	13.8	480	6624
SAP5	13.9	480	6672
SAP6	14.8	480	7104
SAP7	19.5	480	9360
SAP8	20.2	480	9696
SAP9	17.2	480	8256
SAP10	20	480	9600
NC1	21.5	480	10320
NC2	21	480	10080
NC3	18.8	480	9024

NC1–NC3 are control group patients 1–3; SAP1–SAP10, experimental group patients 1–10.

Table S2: Data for all proteins and peptides obtained by data-independent acquisition (DIA)-based proteomic analysis. All proteins and all peptides represent all the proteins and peptides obtained by DIA-based proteomic analysis, respectively. Description of all proteins list header: AccessionID, the first protein ID given in the UniProt database; ProteinAccessions, number of the protein sequence given in the UniProt database; Qvalue, false discovery rate; Genes, gene name given in the UniProt database; ProteinDescriptions, functional description of proteins in the UniProt database based on the protein sequence; ProteinNames, protein name given in the UniProt database. Description of all peptides list header: PEP.StrippedSequence, amino acid sequence of the peptide; PEP.IsProteinGroupSpecific, whether it is the only peptide segment (true or false); EG.PrecursorId, the ID number of Precursor; PG.ProteinGroups, the proteome to which it belongs; PG.ProteinAccessions, login number of the protein to which it belongs; PG.Genes, gene name; PG.ProteinDescriptions, functional description of proteins in database based on protein sequences; PG.ProteinNames, protein name.

The content of this Table S2 is an Excel file. Please check the attached file named Table S2.xlsx

Table S3. Baseline characteristics of the remaining 83 patients with acute pancreatitis

Features/Groups	Non-SAP (n=46)	SAP (n=37)	Statistical result	*P* value
Sex，n (%)			χ^2=0.180	0.671[a]
Male	27 (58.70)	20 (54.05)		
Female	19 (41.30)	17(45.95)		
Age (years)	41.09±9.45	42.76±11.31	*t*=-0.733	0.466[b]
BMI (kg/m^2)	28.04±5.35	29.50±4.47	*t*=-1.328	0.188[b]
SBP (mmHg)	134.09±14.42	130.65±8.24	*t*=1.364	0.177[b]
DBP (mmHg)	81 (76.75-86.25)	87 (83.50-94)	*z*=-3.086	0.002[c]
TC (mmol/L)	8 (6-9)	8 (7-10.5)	*z*=-1.244	0.213[c]
TG (mmol/L)	13.93±4.52	15.49±3.84	*t*=-1.661	0.101[b]
Glu (mmol/L)	7.17±2.76	9.90±3.59	*t*=-3.806	<0.001[b]
IL-1 (pg/mL)	118.19±17.19	144.42±19.55	*t*=-6.498	<0.001[b]
PCT (ng/mL)	5.43±2.85	11.10±5.53	*t*=-5.661	<0.001[b]
WBC (× 10^9/L)	16.47±5.84	12.67±3.80	*t*=3.573	0.001[b]
Amylase (U/L)	380.70±96.95	497.53±136.22	*t*=-4.558	<0.001[b]

BMI, body mass index; SBP, systolic blood pressure; DBP, diastolic blood pressure; TC, total cholesterol; TG, triglycerides; Glu, glucose; IL-1, interleukin-1; PCT, procalcitonin; WBC, white blood cell; SAP, severe acute pancreatitis. The *P* value was obtained for comparison of the groups with following tests: [a]chi-square test; [b]Unpaired Student’s *t*-test; [c]Mann-whitney U test.
